# Editorial: Advances in hearing loss, tinnitus, and vertigo: mechanisms and treatment

**DOI:** 10.3389/fneur.2023.1344956

**Published:** 2024-01-03

**Authors:** Lidong Zhao, Wei Sun, Heng-Wai Yuen, Michael C. F. Tong

**Affiliations:** ^1^Senior Department of Otolaryngology Head and Neck Surgery, Chinese PLA General Hospital; State Key Laboratory of Hearing and Balance Science; National Clinical Research Center for Otolaryngologic Diseases; Key Laboratory of Hearing Science, Ministry of Education; Beijing Key Laboratory of Hearing Impairment Prevention and Treatment; Chinese PLA Medical School, Beijing, China; ^2^Department of Communicative Disorders and Sciences, South Campus, University at Buffalo, New York, NY, United States; ^3^Changi General Hospital, Singapore, Singapore; ^4^Department of Otorhinolaryngology, Head and Neck Surgery, The Chinese University of Hong Kong, Hong Kong, China

**Keywords:** neuro-otologic diseases, pathogenesis, hearing loss, tinnitus, vertigo

Neuro-otologic diseases refer to conditions affecting the cochlea, vestibule, and related nervous system, resulting in symptoms like hearing loss, tinnitus, and vertigo. This group of diseases encompasses various conditions including presbycusis, sudden deafness, drug-induced deafness, noise-induced deafness, hereditary deafness, subjective and objective tinnitus, benign positional paroxysmal vertigo, and Meniere's disease, all of which have a high prevalence and often lead to disability, anxiety, depression, sleep disorders, as well as significant reduction in quality of life.

In this Research Topic, we aim to enhance our understanding of the mechanisms underlying various neuro-otologic diseases. We warmly welcome the submission of Original Articles, Reviews, profiles, commentaries, and perspectives discussing molecular, cellular, and neurologic mechanisms, functional imaging, treatment studies, and clinical trials related to all types of neuro-otologic diseases.

The present Research Topic includes seven articles, comprising a multigenerational cohort study, two computer-aided modeling studies, a retrospective cross-sectional study, a Mendelian and multicentral randomized study, and one review article. These contributions are authored by a collective of 51 researchers from five different countries. They provide valuable insights and diverse research ideas, significantly advancing our understanding of diseases within this domain.

- A team of researchers led by Qin et al. conducted a study to assess the viability of sound conduction without relying on the tympanic air cavity. They explored the possibility of replacing the original cavity with a tympanic vibrating material using finite element analysis. Their results indicated that the shell tympanic vibrating material exhibited superior vibration conduction compared to solid or porous materials. The amplitude of vibration decreased as the elastic modulus of the tympanic vibrating material increased. These findings suggest that substituting the tympanic air cavity with a tympanic vibrating material is a feasible approach.
- Rademaker et al. conducted a study with the aim of developing and internally validating a prediction model for tinnitus experience in a representative sample of the Dutch general population. To accomplish this, they utilized data from the Dutch Lifelines Cohort Study and employed elastic net logistic regression to create a multivariable prediction model. The final model included nine variables: sex, hearing aids, hearing limitations, arterial blood pressure, quality of sleep, general health, symptom checklist of somatic complaints, cardiovascular risk factors, and age. This finding suggests that the model developed by the authors can effectively predict the presence of tinnitus in the studied multigenerational cohort of the Dutch general population.
- Yoon et al. aimed to investigate the impact of a hearing aid policy change in 2015 on the prevalence of hearing loss in the country. They observed a notable increase in both hearing aid prescriptions and registrations for hearing disability following the subsidy increase, which subsequently influenced the prevalence of hearing loss. Based on their findings, the authors emphasized the need for caution when studying the prevalence of hearing loss over medium to long-term periods, given the influence of these factors.
- Zhou et al. noticed that the causal relationship between inflammatory markers and idiopathic sudden sensorineural hearing loss (ISSHL) has not been established. They discovered a significant causal link between genetic susceptibility to C-reactive protein (CRP) levels and the occurrence of ISSHL. This Mendelian randomization study provides compelling causal evidence that CRP is indeed a risk factor for ISSHL. On the other hand, it suggests that TNF-α and fibrinogen do not contribute to an increased risk of developing ISSHL.
- Using CT images of the temporal bone of patients with large vestibular aqueduct syndrome (LVAS), Chen et al. created 3D numerical models and fluid-solid coupling models of the inner ear. With the help of finite element analysis, the authors analyzed the physiological features and pathophysiology of LVAS from a biomechanical perspective. Based on their findings, the authors concluded that the CT images of the temporal bone commonly used in clinical settings could be utilized to establish a comprehensive 3D numerical model of the inner ear, including the VA. They also noted that the VA played a role in limiting the influence of cerebrospinal fluid pressure and that the larger the VA, the less significant the limiting effect on the pressure.
- In a multicenter randomized study, Imai et al. compared the efficacy of two treatment methods, the Epley maneuver (EM) and repeated Dix-Hallpike (DH) tests, in eliminating positional nystagmus in patients with posterior canal benign paroxysmal positional vertigo (pc-BPPV). The results indicated that repeated DH tests were non-inferior to the EM in terms of eliminating positional nystagmus after 1 week in patients with pc-BPPV. Even the disintegration of otoconial debris alone had a therapeutic effect for pc-BPPV because disintegrated otoconial debris can either dissolve in the endolymph or be expelled from the posterior canal through daily activities.
- Bhat et al. conducted a comprehensive review exploring the potential of targeting endocannabinoid system (ECS) components as neuroimmune therapeutic strategies for tinnitus. The authors emphasized the importance of considering gut microbiota dysbiosis, neuroinflammatory mediators, plasma metabolomics biomarkers, as well as audiological and vestibular symptoms following SARS-CoV-2 infection. They believe that such attention and investigation can provide further insights into the interplay between the gut and brain, contributing to our understanding of the pathophysiology of tinnitus and the identification of potential therapeutic targets. This review serves as a valuable resource for researchers and healthcare professionals interested in the neuroimmune aspects of tinnitus and opens avenues for future investigations in this field.

In summary, the articles and review presented in this Research Topic of the Journal represent significant advancements in our understanding of the mechanisms underlying inner ear diseases. The insights gained from these studies promise to have a profound impact on our understanding of inner ear diseases and may pave the way for novel approaches and interventions. By shedding light on the intricate mechanisms at play, these contributions have the potential to revolutionize our diagnostic and therapeutic strategies, ultimately improving patient outcomes.

## Author contributions

LZ: Writing—original draft, Writing—review & editing. WS: Writing—review & editing. H-WY: Writing—review & editing. MT: Writing—review & editing.

